# Anemonin attenuates osteoarthritis progression through inhibiting the activation of IL‐1β/NF‐κB pathway

**DOI:** 10.1111/jcmm.13227

**Published:** 2017-06-23

**Authors:** Zuqiang Wang, Junlan Huang, Siru Zhou, Fengtao Luo, Wei Xu, Quan Wang, Qiaoyan Tan, Liang chen, Jun Wang, Hangang Chen, Lin Chen, Yangli Xie, Xiaolan Du

**Affiliations:** ^1^ Department of Rehabilitation Medicine Center of Bone Metabolism and Repair State Key Laboratory of Trauma, Burns and Combined Injury Trauma Center Research Institute of Surgery Daping Hospital Third Military Medical University Chongqing China

**Keywords:** anemonin, cartilage, osteoarthritis, NF‐κB

## Abstract

The osteoarthritis (OA) progression is now considered to be related to inflammation. Anemonin (ANE) is a small natural molecule extracted from various kinds of Chinese traditional herbs and has been shown to inhibiting inflammation response. In this study, we examined whether ANE could attenuate the progression of OA *via* suppression of IL‐1β/NF‐κB pathway activation. Destabilization of the medial meniscus (DMM) was performed in 10‐week‐old male C57BL/6J mice. ANE was then intra‐articularly injected into joint capsule for 8 and 12 weeks. Human articular chondrocytes and cartilage explants challenged with interleukin‐1β (IL‐1β) were treated with ANE. We found that ANE delayed articular cartilage degeneration *in vitro* and *in vivo*. In particular, proteoglycan loss and chondrocyte hypertrophy were significantly decreased in ANE ‐treated mice compared with vehicle‐treated mice. ANE decreased the expressions of matrix metalloproteinase‐13 (MMP13), A disintegrin and metalloproteinase with thrombospondin motifs 5 (ADAMTS5), collagen X (Col X) while increasing Aggrecan level in murine with DMM surgery. ANE treatment also attenuated proteoglycan loss in human cartilage explants treated with IL‐1β *ex vivo*. ANE is a potent protective molecule for OA; it delays OA progression by suppressing ECM loss and chondrocyte hypertrophy partially by suppressing IL‐1β/NF‐κB pathway activation.

## Introduction

OA is one of the most prevalent joint disorders, characterized by articular cartilage degeneration, joint pain and functional impairment 1. Thus far, there are few effective disease‐modifying treatments for OA. The final option is usually the total joint replacement surgery 2, 3. Therefore, finding effective therapies to attenuate the progression of OA in patients is urgent. Mechanism‐based therapies are the important research directions in future.

Although the precise pathogenesis of OA is still not fully obvious, inflammatory response is now considered to be involved in the development and progression of OA 4, 5. Secreted inflammatory factors such as pro‐inflammatory cytokines are vital mediators for the disturbed metabolism in OA 6, 7. The catabolic effects of IL‐1β are mainly mediated *via* the activation of several signalling pathways including the p38 mitogen‐activated protein kinase and c‐Jun N‐terminal kinase, and most importantly, nuclear factor κB (NF‐κB) signalling is activated 6. NF‐κB signalling not only regulates the expressions of multiple inflammatory genes but also participates in the expression or activity induction of matrix‐degrading enzymes in OA 8. Chen and colleagues have shown that Ad‐siRNA^NF−κBp65^ attenuated the progression of OA in a rat surgery model 9. This evidence indicates that therapies blunting inflammatory response are appropriate candidates for OA treatment.

In traditional Chinese medicine, a variety of herbs including Clematis have been used to alleviate OA symptoms effectively 10, 11, 12. Recent clinical trial suggested that extracted mixture of Clematis exerted protective effects on OA 13. However, the precise mechanism and responsible molecules for this effect are not obvious presently. We noticed that ANE (dilactone of cyclobutane‐1,2‐diol‐1,2‐diacrylic acid) is a small natural molecule isolated from herbs including Clematis 14. ANE structure was identified in a previous study (Fig. S1) 15. In previous studies, ANE showed neuroprotective effect and inhibited pigmentation synthesis in human melanocytes 15, 16. ANE also has been reported to exhibit anti‐inflammatory properties *via* inhibiting the production of nitric oxide induced by lipopolysaccharide (LPS) in endothelial cells 17. So we suggested that ANE may have therapeutic effect on OA partially though inhibiting inflammatory response.

In this study, we investigated the effect of ANE on OA progression. We found that ANE can attenuate the articular cartilage degeneration in murine DMM model and human cartilage explants in part by inhibiting the activation of IL‐1β/NF‐κB pathway.

## Materials and methods

### Surgical mouse model of OA

10‐week‐old male C57BL/6J (WT) mice were purchased from animal facility of the Daping Hospital (Chongqing, China). DMM surgery was performed on the right knee joints of 10‐week‐old male mice according to previous study 18. The mice were first anesthetized (1% pentobarbital sodium), joint capsule was incised, and then, the medial meniscotibial ligament was sectioned with microsurgical scissors. As a control, surgery was performed on left knee joints but the ligaments were remained intact and regarded the joint as ‘sham joints’. All mice were allowed to move freely in the cages after DMM surgery. After surgical OA induction, the animals were randomly divided into two groups, namely ANE treatment group and vehicle (control) group (*n* = 14 per group). Mice were killed at 8 and 12 weeks after DMM surgery. All mice were maintained in the animal facility (specific pathogen free) of the Daping Hospital. All experiments were performed according to protocols approved by the Laboratory Animal Welfare and Ethics Committee of Third Military Medical University (Chongqing, China).

### ANE treatment

ANE was bought from ChromaDex Company (Irvine, CA, USA) and reconstituted in 50% (w/v) 2‐hydroxypropyl‐β‐cyclodextrin (Sigma‐Aldrich, St. Louis, MO USA) in PBS. From the second day after surgery in mice, ANE of 2 mg/kg bodyweight or equivalent volume of vehicle (50% 2‐hydroxypropyl‐β‐cyclodextrin and PBS) was injected intra‐articularly once a week for 8 weeks. These mice were killed at 8 and 12 weeks after DMM.

### Sample preparation

Mice were killed by CO2 inhalation. Specimens consisting of whole femora and tibiae were removed, cleaned of muscle and ligament, fixed in 4% paraformaldehyde (PFA) (Sigma‐Aldrich) and stored at 4°C. Then, they were decalcified in 20% formic acid and embedded in paraffin.

### Histologic assessment of articular cartilage degeneration

Histologic scoring for mouse cartilage degeneration was performed according to OARSI‐recommended scoring system reported by Glasson 19. Briefly, three 5‐μm sections were placed on each slide, with each joint fully harvested in 10‐slide intervals. Sections of the joints at every 50 μm (10 slides per joint) were stained with Safranin O–Fast Green to assess the degeneration of articular cartilage. The intensity of Safranin O staining in the growth plate of the femora and tibiae was used as an internal control between batches.

The severity of cartilage degeneration was evaluated for a summed score (sum of the four highest scores in all slides) and as a maximal score for the medial femora and medial tibiae separately within each section. All investigators were blinded to allocation during experiments and outcome assessment. Scoring was carried out by three independent investigators.

### Immunohistochemistry

Decalcified bone sections were deparaffinized with xylene, and endogenous peroxidase activity was quenched with 3% H_2_O_2_ for 15 min., followed by antigen retrieval with trypsinization for 15 min. Then, sections were blocked with normal goat serum for 30 min. and incubated at 4°C overnight with primary antibody followed by the biotinylated secondary antibody and horseradish peroxidase‐conjugated streptavidin–biotin staining. Immunoreactivity was visualized with a 3, 3′‐diaminobenzidine tetrahydrochloride kit (ZSGB‐BIO, Beijing, China) followed by counterstaining with methyl green. Primary antibodies against the following proteins were used: collagen II (1:400; Chondrex, Redmond, WA, USA), collagen X (1:200; Abcam, Cambridge, MA, USA), Aggrecan (1:200 Abcam), MMP13 (1:200; PeproTech, Chicago, IL, USA), ADAMTS5 (1:200; Abcam), cleaved caspase‐3 (1:100; Boster, China) and phosphor‐P65 (1:200; CST, Danvers, MA, USA). The number of immunoreactive cells in sections was counted using Image‐Pro Plus 5.1 (Media Cybernetics, Rockville, MD, USA).

### Human articular cartilage explant culture

Human articular cartilage tissue harvested from patients undergoing total joint replacement surgery at Daping Hospital because of traffic accidents was collected (*n* = 10, Mankin score: 0–2, average age: 37 years). Samples were collected with the approval by the Institutional Review Board and Ethics Committee of Daping Hospital and consent from the patients and families**.** For cartilage explant culture, full‐thickness cartilage tissue was cut into pieces of 5 mm. Following 48 hrs of culture in Dulbecco's modified Eagle's medium/F‐12 containing 10% foetal bovine serum and 50 units/ml of penicillin and streptomycin, the explants were treated with IL‐1β (10 ng/ml) (PeproTech), ANE (10 μM) and ibuprofen (10 μM)(Sigma‐Aldrich) for 4 days under serum‐free conditions with ITS (insulin–transferrin–selenium; Sigma‐Aldrich) according to previous studies 20, 21, 22, 23. Following 4‐day culture, the medium was collected and the explants were fixed in 4% paraformaldehyde.

### Isolation and culture of human articular chondrocytes

Human articular chondrocytes were isolated from the healthy articular cartilage tissues according to previously described method 24. Isolated chondrocytes were plated into six‐well plates at a density of 1 × 10^6^ cells/well and cultured in Dulbecco's modified Eagle's medium/F‐12 (HyClone, Logan, UT, USA) containing 10% foetal bovine serum (Gibco/Life Technologies, Carlsbad, CA, USA) and 50 units/ml of penicillin and streptomycin (HyClone). At 80% confluence, human chondrocytes were cultured under serum‐free conditions for 24 hrs. The chondrocytes were incubated with ANE (10 μM, ChromaDex, Irvine, CA, USA) for 1 hr before 10 ng/ml IL‐1β treatment for 4 hrs and 24 hrs as previously described 17.

### Cell line culture and treatment

Rat chondrosarcoma (RCS) cells were cultured in DMEM/F12 (1:1), supplemented with 5% FBS. siRNA^NF−κBp65^ (RiboBio, Guangzhou, China) (30 nM) transfection was performed with Lipofectamine^®^ 3000 (Invitrogen, Waltham, MA, USA) in accordance with the manufacturer's protocols.

### Dimethylmethylene blue assay

The DMMB dye binding assay was performed to measure glycosaminoglycan (GAG) release of the cultured explants 25. Briefly, 250 μl of DMMB reagent was added to 40 μl of culture medium, and the absorbance was examined at 525 nm. We used different concentrations of chondroitin sulphate (Sigma‐Aldrich) to plot a standard curve, and the amount of GAG released was determined. GAG released into the medium was normalized as mass of GAG per millilitre (ml) of culture medium.

### RNA isolation and quantitative RT‐PCR (qPCR)

Total RNA was isolated from human chondrocyte cultures using the TRIzol reagent (Invitrogen). Real‐time qPCR was performed using Mx3000p PCR machine (Stratagene, Santa Clara, CA, UAS) and SYBR Premix Ex TaqTM kit (Takara, Shiga, Japan). All samples were measured in triplicate, and the GAPDH was amplified as internal control. The primer sequences for qPCR were shown in Fig. S4.

### Western blotting

Human articular chondrocyte cultures were extracted with RIPA lysis buffer containing protease inhibitors (Roche, West Sussex, United Kingdom). Proteins were resolved by 10% or 12% sodium dodecyl sulphate polyacrylamide gel electrophoresis and transferred to a polyvinylidene difluoride membrane (Millipore, Billerica, MA, USA). After being blocked with 5% non‐fat milk in Tris‐buffered saline–Tween buffer, the membrane was probed with primary antibodies specific for IL‐1β (Beyotime), p‐IKK‐α/β (CST), p‐P‐65 (CST), MMP13 (Millipore) and Aggrecan (Abcam) followed by secondary antibodies. The signal was detected with chemiluminescence (Pierce) according to the manufacturer's protocols. β‐actin (Sigma‐Aldrich) was applied to normalize the protein expression levels.

### X‐ray and micro‐CT analysis

X‐ray images were obtained using MX‐20 Cabinet X‐ray system (*n* = 6 per group) (Faxitron X‐Ray, Tucson, AZ, USA). Ionic contrast‐enhanced agent was used to observe the integrity of articular cartilage with Scanco vivaCT (Scanco Medical, Brüttisellen, Switzerland). The femurs were immersed in 40% Hexabrix and 60% 1XPBS for 1 hr at 37°C according to previous study 26. Serial 21‐mm tomographic images were acquired at the condition of 45 kV and 112 mA, and the cartilage was defined as 143 threshold levels. Tibiae were scanned and analysed for subchondral bone density according to our previous study 27. Serial 12.5‐μm 2D images were acquired at 70 kV and 113 mA. 2D images were obtained, and region of interest (ROI) in subchondral bone was defined as medial tibial trabecular epiphyses and subchondral bone plate thickness (*n* = 7 per group).

### Statistical analysis

The numeric data were expressed as 95% confidence intervals. One‐way analysis of variance (anova) was used for multifactorial comparisons in this study. Homogeneity of variance was tested, and then, the differences between groups were assessed by *post hoc* multiple comparisons. Data were analysed by GraphPad Prism v.6.01 software (GraphPad Inc., La Jolla, CA, USA). *P* < 0.05 was considered statistically significant.

## Results

### Intra‐articular injection of ANE delays the progression of OA in DMM model

The side effects of ANE such as vomit and diarrhoea were not observed in mice following local intra‐articular injection of ANE. No gross abnormalities in the knee joints were observed in either the ANE‐treated or vehicle‐treated mice (Fig. S2).

**Figure 1:**
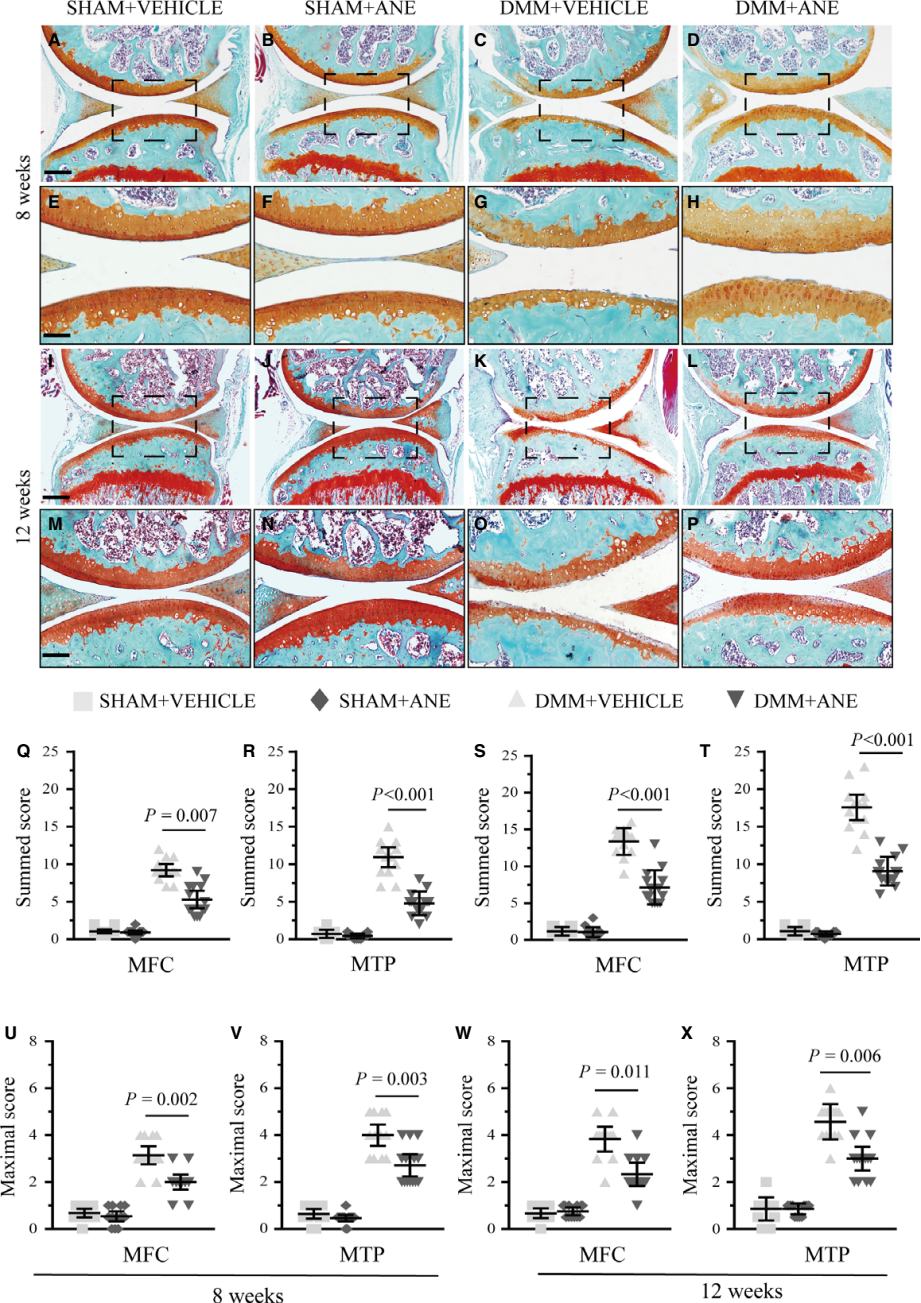
Effects of anemonin on cartilage degradation in mouse articular cartilage at 8 and 12 weeks after DMM. After DMM surgery or sham operation, mice were intra‐articularly injected with vehicle (50% 2‐hydroxypropyl‐β‐cyclodextrin) or anemonin (2 mg/kg bodyweight) during following 8 weeks and 12 weeks (*n* = 14 per group). (**A**–**P**) Knee joints were harvested at 8 weeks and 12 weeks after surgery and analysed histologically by Safranin O–fast green staining. Representative images were shown. (**Q**–**X**) Summed and maximal histologic scores for cartilage structure damage were evaluated by OARSI‐recommended scoring system in mice with or without anemonin treatment after DMM surgery at 8 weeks and 12 weeks. MFC: medial femoral condyle; MTP: medial tibial plateau. Scale bar: 200 μm (**A**–**D** and **I**–**L**); 100 μm (**E**–**F** and **M**–**P**). Data are expressed as the mean (symbols) ± 95% confidence intervals (error bar).

To investigate the effects of ANE on progression of OA histologically, we performed intra‐articular injection of ANE in mice after DMM. The optimal dose (2 mg/kg bodyweight) was identified by testing several concentrations of ANE (0.5, 2 and 5 mg/kg) (Fig. S2 and S3). Lower concentration (0.5 mg/kg) had weak protective effects, and higher concentration (5 mg/kg) led to severer proteoglycan loss than intermediate concentration (2 mg/kg) in articular cartilage after DMM surgery. We thus used relatively optimal dose (2 mg/kg) for the following experiments *in vivo*. We found at 8 weeks after DMM surgery (Fig. 1A–H), Safranin O staining showed preservation of proteoglycan and decreased chondrocyte hypertrophy in the ANE‐treated group compared to vehicle‐treated group. Moreover, at 12 weeks after DMM surgery, the vehicle‐treated mice had more severe phenotypes including articular fibrillation and loss of articular cartilage tissue and increased chondrocyte hypertrophy in superficial zone (Fig. 1I–P), which is similar to those of human OA.

OARSI histologic scoring system was applied to quantitatively analyse the cartilage degeneration after DMM surgery 19. The summed and maximal OARSI score of femurs and tibiae demonstrated that ANE‐treated mice had a significantly lower OARSI score than vehicle‐treated mice at 8 and 12 weeks after DMM surgery (Fig. 1Q–X). However, the summed OARSI score in the ANE‐treated mice at 12 weeks was increased compared to the score at 8 weeks.

### ANE attenuates the cartilage degeneration but not subchondral bone remodelling in DMM model by micro‐CT analysis

**Figure 2:**
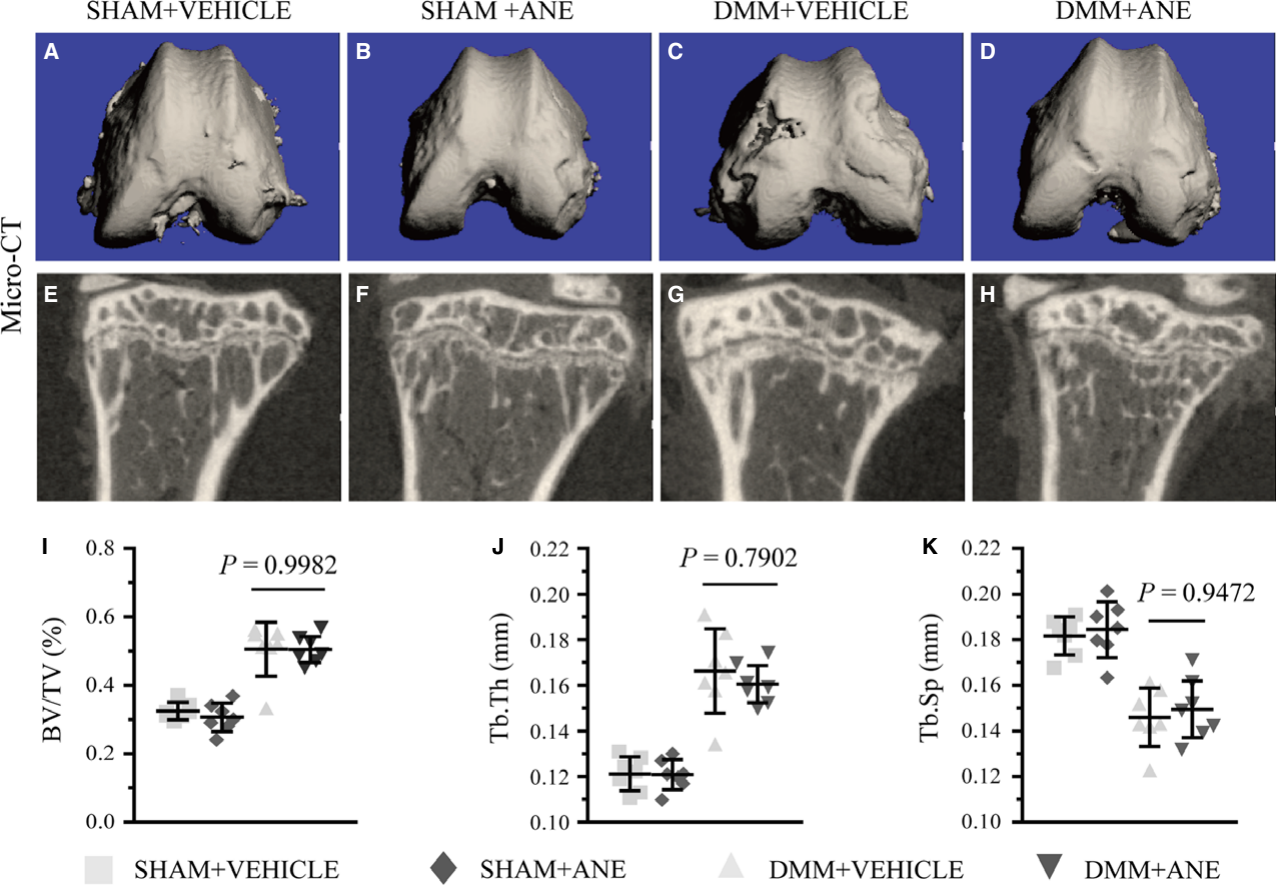
Micro‐CT analysis of ANE effects on tibia after DMM surgery. (**A**–**D**) Contrast agent‐enhanced tomography of articular cartilage in mice after DMM surgery. (**E**–**H**) Micro‐CT 2D reconstructions of tibial subchondral bone in mice after DMM surgery or sham operation.(**I**) Bone volume/total volume (BV/TV), (**J**) trabecular thickness (Tb.Th) and (**K**) trabecular separation (Tb.Sp) were measured in the subchondral bone of tibia of vehicle‐treated and anemonin‐treated mice at 8 weeks after DMM surgery or sham operation (*n* = 7 per group). Data are expressed as the mean (symbols) ± 95% confidence intervals (error bar).

To observe the damage severity of articular cartilage directly after DMM surgery, we performed the contrast agent‐enhanced cartilage computed tomography at 8 weeks after DMM surgery 26. The results revealed that articular cartilage structure was much less severely damaged in ANE‐treated mice (2 mg/kg bodyweight) compared to vehicle‐treated mice (Fig. 2A–D). In addition, micro‐CT analysis showed that the relative bone volume fraction (bone volume/total volume, BV/TV) and trabecular thickness (Tb.Th) were increased by 63.75% and 33.31%, respectively, and trabecular separation (Tb.Sp) was decreased by 17.76% in the tibial subchondral bone of DMM mice compared to that of sham controls (Fig. 2E–H). However, no significant difference in BV/TV and Tb.Th of the tibial subchondral bone was observed between vehicle‐treated DMM mice and ANE‐treated DMM mice (Fig. 2I–K). These results suggested that intra‐articular injection of ANE has protective effects on articular degeneration, it while shows no significant influence on subchondral bone remodelling in the DMM model.

### ANE decreases the expressions of MMP13 and collagen X in DMM model

**Figure 3:**
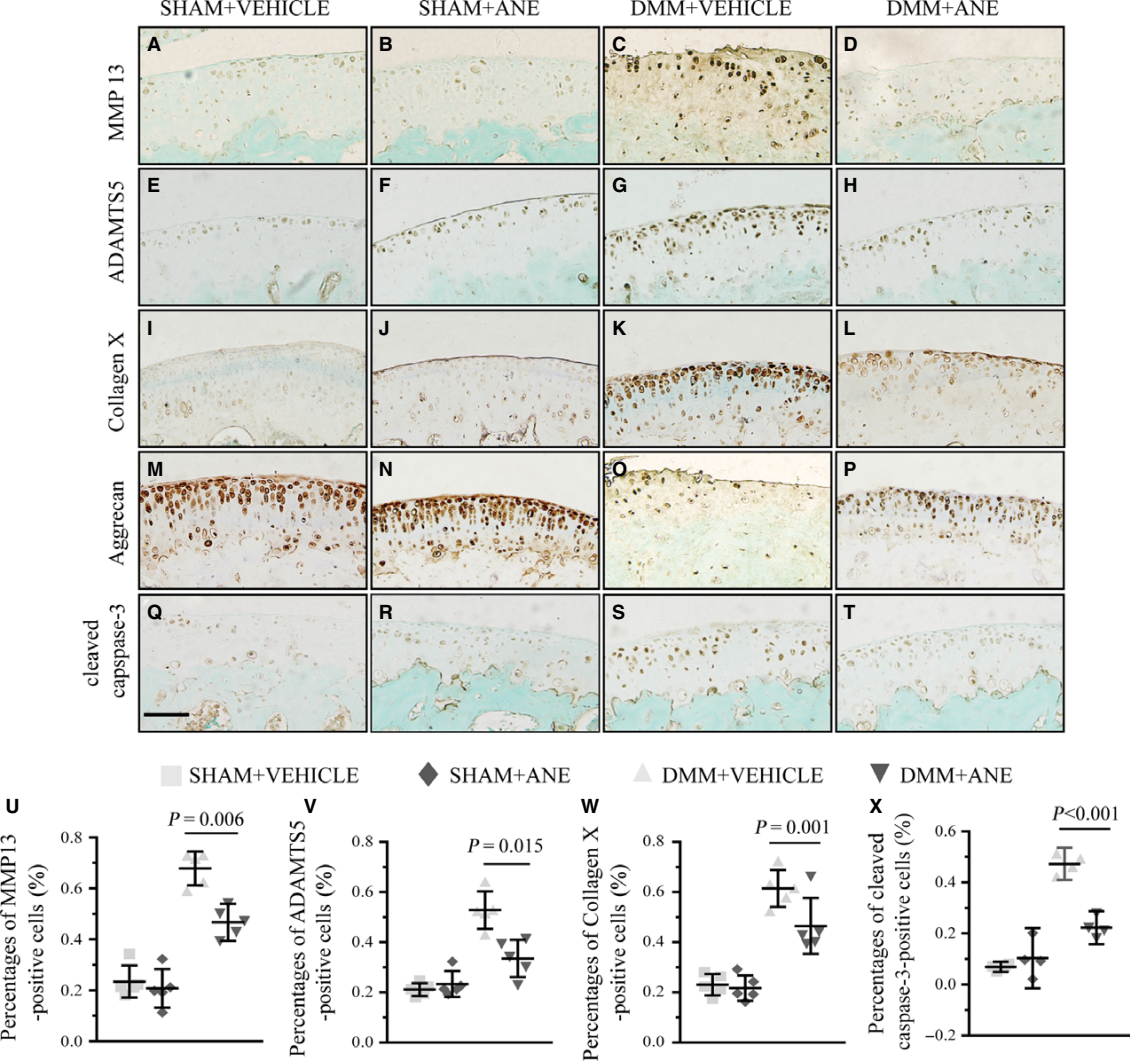
The effects of anemonin treatment on the homoeostasis of articular cartilage after DMM surgery. Sections of articular cartilage from the mice with or without anemonin treatment after 8 weeks of DMM surgery and sham operation were analysed by immunohistochemical staining (*n* = 5 per group). IHC stainings of (**A**–**D**) MMP13, (**E**–**H**) ADAMTS5, (**I**–**L**) collagen X, (**M**–**P**) Aggrecan and (**Q**–**T**) cleaved caspase‐3 were performed in mice after DMM surgery. (**U**–**X**) The ratios of immunoreactive positive cells, MMP13 (**U**), ADAMTS5 (**V**), collagen X (**W**) and cleaved caspase‐3 (**X**) were analysed. Scale bar: 50 μm (**A**–**T**). Data are expressed as the mean (symbols) ± 95% confidence intervals (error bar).

To explore the mechanisms underlying the delayed progression of cartilage degradation in ANE‐treated mice with DMM, we performed IHC staining to examine the pathological changes in articular cartilage. MMP13 and ADAMTS5 immunoreactivities were decreased, while those of Aggrecan were increased in ANE‐treated DMM mice compared to vehicle‐treated DMM mice (Fig. 3A–D, E–H, M–P, U and V). In addition, ANE treatment significantly decreased the expression of collagen X (Fig. 3I–L and W).

Chondrocyte apoptosis is closely related to OA progression 28, 29, and we examined chondrocyte apoptosis by IHC staining of cleaved caspase‐3. The number of cleaved caspase‐3‐positive cells in the articular cartilage was markedly decreased by 32% in the ANE group compared to the vehicle group at 8 weeks after DMM surgery (Fig. 3Q–T and X).

### ANE attenuates degeneration of human articular cartilage in an *ex vivo* model

**Figure 4:**
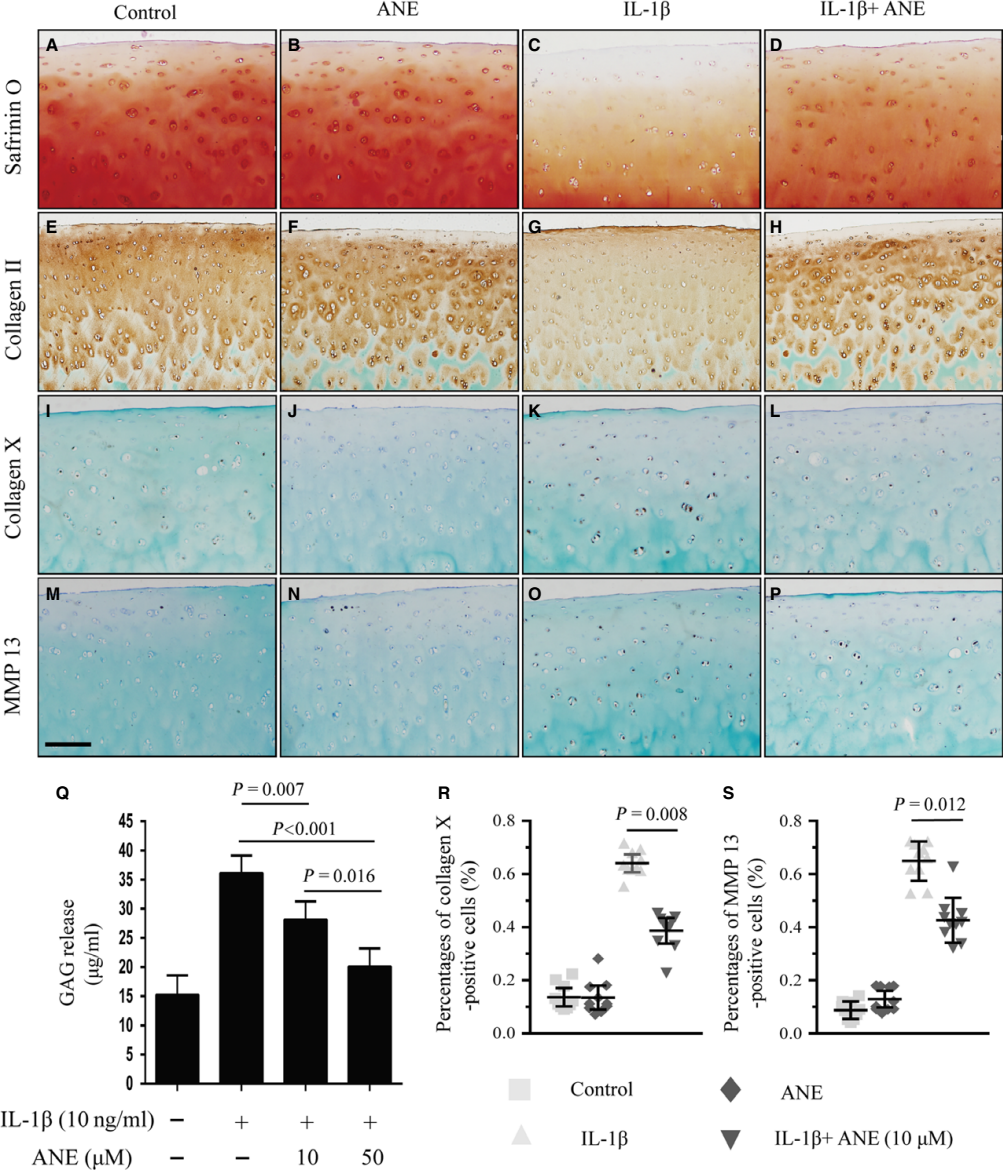
Effects of anemonin on human articular cartilage *ex vivo*. The full‐thickness human cartilage explants were obtained from adult human joint tissues (*n* = 10, Mankin score 0–2). Cartilage explants were cultured in the absence or presence of IL‐1β (10 ng/ml) and anemonin (10 μM) for 4 days. (**A**–**D**) 5‐μm paraffin sections were stained with Safranin O–fast green to examine the cartilage proteoglycan loss. (**E**–**H**) Immunohistochemical staining for collagen II, (**I**–**L**) collagen X and (**M**–**P**) MMP13 in human articular cartilage. (**R**) Collagen X‐ and (**S**) MMP13‐positive cells in human articular cartilage were counted. (**Q**) Culture medium were collected, and the amount of GAG release into the medium was quantified by DMMB assay. GAG released into the medium was normalized as mass of GAG per millilitre (ml) of culture medium. Scale bar: 200 μm (**A**–**P**). Data are expressed as the mean (symbols) ± 95% confidence intervals (error bar).

IL‐1β has been well known to play a key role in the degradation of articular cartilage by inhibiting ECM synthesis and accelerating cartilage breakdown. To further investigate the effects of ANE on human articular cartilage, we used IL‐1β‐induced OA model of human articular cartilage *ex vivo*. Safranin O staining showed that ANE significantly attenuated IL‐1β‐induced loss of proteoglycan (Fig. 4A–D). IHC staining further revealed notably increased expression of collagen II in ANE‐treated cartilage explants compared to vehicle‐treated cartilage explants (Fig. 4E–H). ANE also decreased the number of MMP‐13 (Fig. 4M–P and S)‐ and type X collagen (Fig. 4I–L and R)‐positive cells by 38.5% and 43.7%, respectively, which were consistent with the observation in ANE‐treated mice with DMM surgery.

We also collected culture medium to analyse the glycosaminoglycan (GAG) release with DMMB assay. The results showed that ANE treatment significantly decreased the release of GAG into the culture medium from IL‐1β‐stimulated human articular cartilage tissues (Fig. 4Q). In addition, we compared the protective effects between ANE and ibuprofen (a classical non‐steroidal anti‐inflammatory drug) in IL‐1β‐induced OA model. The results showed that both ANE and ibuprofen alleviated IL‐1β‐induced proteoglycan loss (Fig. S5).

### ANE decreases the expressions of catabolic genes in IL‐1β‐treated human articular chondrocytes

**Figure 5:**
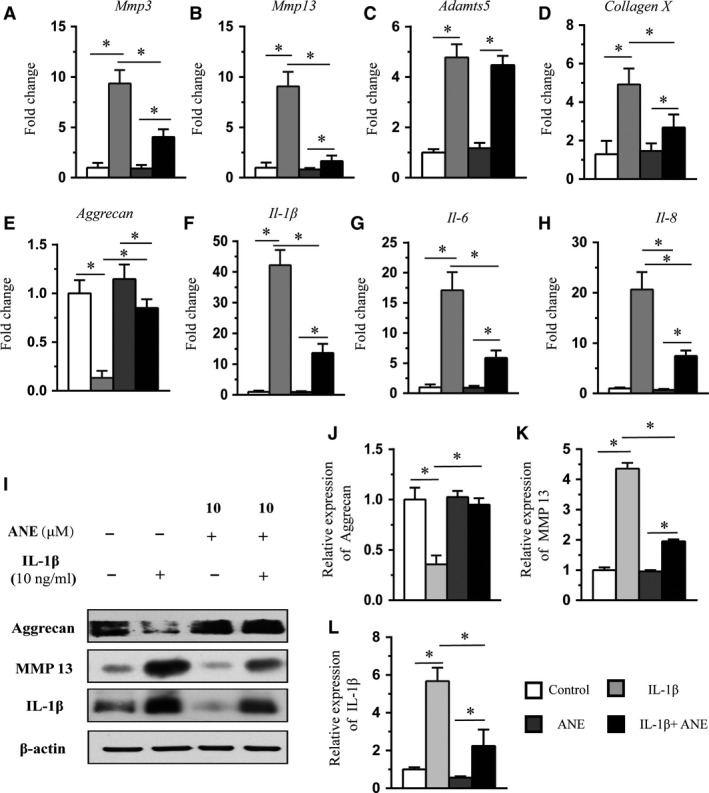
Effects of anemonin on the mRNA levels of catabolic event genes in IL‐1β‐stimulated human articular chondrocytes. (**A**–**H**) Human articular chondrocytes were cultured without serum for 24 hrs and then treated with anemonin (10 μM) for 1 hr, followed by treatment of IL‐1β (10 ng/ml) for 24 hrs. Total RNA was isolated, and levels of mRNAs for the chondrocytes catabolic event markers (*mmp3*,*mmp13* and *collagen X*), articular chondrocyte marker (*Aggrecan*) and pro‐inflammatory cytokines (*Il‐1*,* Il‐6* and *Il‐8*) were detected by RT‐PCR. (**I**) Cell lysates were analysed by Western blot with antibodies specific for MMP13, Aggrecan and IL‐1β. β‐actin was used as a loading control. Data are expressed as the normalized fold expression relative to controls. (**J**–**L**) The signal intensities of Aggrecan, IL‐1β and MMP 13 were quantified using software ImageJ (version 1.47) (*n* = 5). Data are expressed as the mean (symbols) ± 95% confidence intervals (error bar). *P*‐values between groups with * are <0.05.

We next investigated the effect of ANE on the expressions of matrix‐degrading enzymes and pro‐inflammatory factors in human chondrocytes. RT‐PCR results showed that ANE decreased IL‐1β‐mediated up‐regulation of the expressions of *mmp3*,* mmp13* and *collagen 10,* but not *adamts5,* in human chondrocytes. In addition, ANE increased the expression of *Aggrecan* (Fig. 5A–E). Moreover, we also observed that ANE significantly decreased the expressions of inflammatory factors including IL‐1β, IL‐6 and IL‐8 (Fig. 5F–H). Western blotting further demonstrated that ANE significantly decreased the MMP13 and IL‐1β expressions and reversed the decreased expression of Aggrecan (Fig. 5I and J–K). These results demonstrated that ANE inhibited catabolic events in IL‐1β‐stimulated human chondrocytes.

### ANE suppresses NF‐κB signalling pathway by decreasing the phosphorylation of IKKα/β and p65

**Figure 6:**
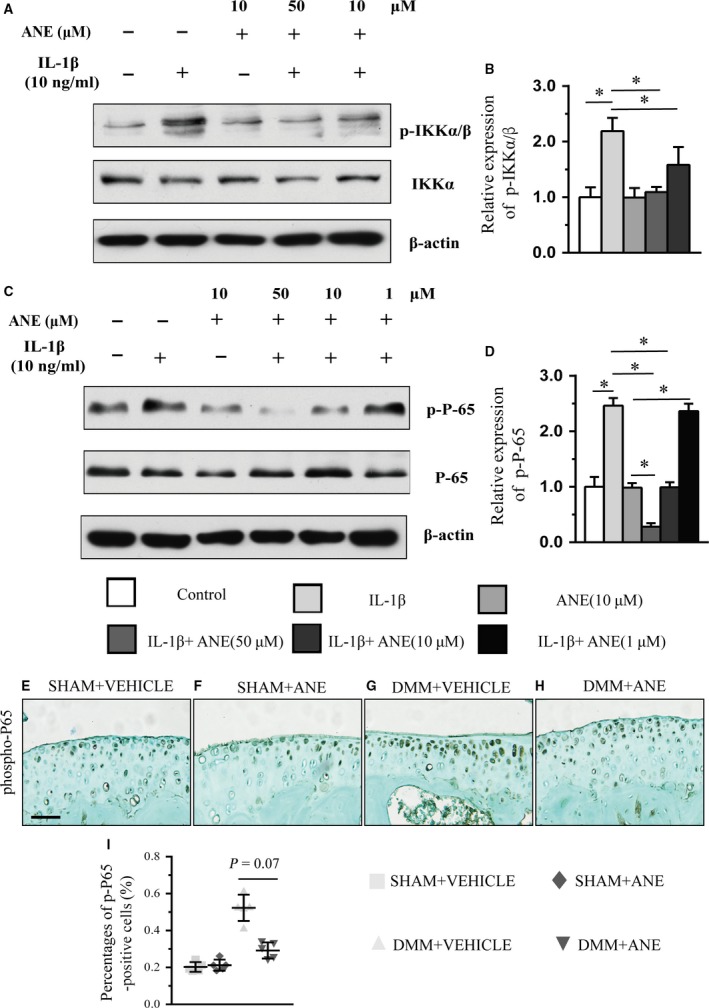
Effects of anemonin on NF‐κB signalling in IL‐1β‐induced human chondrocytes and DMM mouse model. (**A** and **C**) Western blot analysis of the phosphorylation of IKKα/β (**A**) and p65 (**C**) in human chondrocytes treated with the combination of recombinant human IL‐1β and decreasing doses of anemonin for 4 h. (**B** and **D**) The relative expression of protein level of (**B**) p‐IKKα/β and (**D**) p‐P‐65 with anemonin treatment *in vitro*. The knee joints were immunohistochemically stained for (**E**–**H**) phospho‐P65 at 8 weeks after DMM with intra‐articular injection of anemonin or vehicle. The ratios of immunoreactive positive cells. (**I**) phospho‐P65‐positive cells were quantified (*n* = 5 per group). Scale bar: 50 μm (**D**–**G**). Data are expressed as the mean (symbols) ± 95% confidence intervals (error bar). *P*‐values between groups with * are <0.05.

As NF‐κB signalling pathway plays a key role in the up‐regulation of extracellular matrix‐degrading enzymes and chondrocyte hypertrophy during OA progression, we examined whether the protective effects of ANE on OA are related to its regulation of NF‐κB signalling in human articular chondrocytes treated with IL‐1β. We analysed the phosphorylated and total protein levels of IKKα/β and p65 by Western blot. We found that ANE significantly decreased the levels of phosphorylated IKKα/β (Fig. 6A–B) and p65 (Fig. 6C–D) compared to control in a dose‐dependent manner, but revealed no significant difference of total protein of IKKα and p65 between ANE and control groups. Moreover, we performed IHC staining for phospho‐p65 in the articular cartilage at eight weeks after DMM and found that ANE decreased phospho‐p65 level, indicating that ANE may inhibit the NF‐κB signalling in articular cartilage (Fig. 6E–H). Specifically, ANE treatment significantly decreased the number of phospho‐p65‐positive cells by 47.31% compared with vehicle treatment (Fig. 6I). In addition, we employed RCS cell line and transfected RCS cells with siRNA^NF−κBp65^. Western blot analysis showed that both ANE and siRNA^NF−κBp65^ notably inhibited the MMP13 and phosphorylated p65 expressions in IL‐1β‐stimulated RCS cells (Fig. S6). These results indicated that ANE treatment suppressed the phosphorylation of IKKα/β and p65 in OA progression.

## Discussion

OA is one of the most prevalent joint diseases that may eventually lead to physical disability. The pathological mechanism of OA is poorly understood, and there are few effective treatments to delay the development of OA. The development and progression of OA are now considered to be related to inflammation in the early stage 30. Secreted inflammatory factors such as IL‐1β play critical roles in the pathophysiology of OA *via* the activation of a variety of signalling pathways such as nuclear factor κB (NF‐κB). To date, the pharmacological therapies of OA are mainly focused on alleviating pain and inflammation using non‐steroidal anti‐inflammatory drugs (NSAIDs) or other agents 31, which have potential side effect^32^, ^33^. Given that OA is a chronic disease occurring mainly in older population, the safety of medicine for OA treatment is very important. However, the side effects of these medicines seem to be inevitable. Chinese herbs have long been used to alleviate OA in Chinese medicine with less side effects. The small molecule ANE isolated from plant Clematis has been found to clinically alleviate OA symptoms. In the present study, we found that ANE, a plant‐derived natural molecule, attenuated articular cartilage degeneration in part by inhibiting the phosphorylation of p65 subunit of NF‐κB.

Secreted inflammatory molecules such as IL‐1β are the key mediators of the disturbed homoeostasis in OA. Interestingly, IL‐1β also stimulates the production of multiple other inflammatory mediators such as IL‐6 and IL‐8 in OA pathology 34, 35. In our study, ANE decreased the expressions of IL‐1β, IL‐6 and IL‐8 in IL‐1β‐treated human chondrocytes. The pro‐inflammatory factors can be up‐regulated through the activation of NF‐κB signalling, a potential target for OA therapy 8. P65 is a critical active subunit in NF‐κB signalling in multiple cell types 36. Liu found that the percentages of phosphorylated p65 were significantly increased in TMJ‐OA mice 37. Chen showed that p65‐specific siRNA could suppress the induction of IL‐1β and delay the cartilage degradation in OA model in early‐phase 9. Collectively, this evidence indicates that p65 subunit of NF‐κB pathway is highly associated with the development and progression of OA. In this study, we found that ANE attenuated OA progression partially through its inhibition of the phosphorylation of p65 in IL‐1β‐induced human chondrocytes and articular cartilage of mice with DMM surgery. Furthermore, notably mild articular degeneration was found in the ANE‐treated group compared to vehicle‐treated group in both eight weeks and twelve weeks after DMM surgery, but the summed OARSI score in the ANE‐treated mice at 12 weeks was still increased compared to the score at 8 weeks. These results indicated that ANE treatment delayed the progression of OA in DMM mouse model.

Decades of studies have demonstrated that one fundamental feature of OA is the imbalance between anabolic and catabolic metabolism of the extracellular matrix of chondrocytes 1, 38. There are two major types of molecule changes in the OA pathology: up‐regulation of tissue‐destructive enzymes such as MMP3, MMP13 and ADAMTS5, and down‐regulated protein levels in ECM molecules including Aggrecan and collagen II. Multiple studies demonstrated that pro‐inflammatory factors such as IL‐1β could suppress the synthesis of ECM molecules such as Aggrecan and collagen II 7. IL‐1β can also stimulate chondrocytes to release matrix‐degeneration enzymes such as MMP3 and MMP13 39, 40. Furthermore, several studies indicated that p65 subunit of NF‐κB could directly bind to the promoters of some catabolic genes for the cartilage degeneration such as *adamts5* 41. Therefore, p65 subunit of NF‐κB pathway may play a critical role, as a catabolic factor, in the progression of OA. In our study, ANE altered expressions of *mmp3*,* mmp13* and *Aggrecan* in IL‐1β‐treated human articular chondrocytes. We further found that ANE significantly decreased the expression of MMP13 and increased the proteoglycan level in IL‐1β‐stimulated human cartilage explants and articular cartilage of mice with DMM surgery. These findings suggested that ANE may delay the articular cartilage degeneration by suppressing the expressions of cartilage‐destructive enzymes and maintaining the ECM level.

Besides ECM degradation, accelerated hypertrophy of chondrocytes now is also considered to play a vital role in the progression of OA 42. Although the reasons for the accelerated chondrocyte hypertrophy are not fully clarified, NF‐κB signalling has been found to be involved in it 43. NF‐κB pathway could inhibit the chondrogenic differentiation of mesenchymal cells by down‐regulating SOX9 44. Olivotto suggested that the canonical NF‐κB pathway in the chondrocytes *via* IKK signalling plays a critical role in OA development and progression, and excessive IKK activity may be responsible for the abnormal hypertrophy of OA chondrocytes 45. Inhibition of NF‐κB pathway in chondrocytes suppressed the expression of MMP13, an important marker for chondrocyte hypertrophy 46. Thus, this evidence indicated that NF‐κB pathway is closely associated with the abnormal chondrocyte hypertrophy in the progression of OA. In our study, we found that ANE inhibited the phosphorylation of IKKα/β in the IL‐1β‐treated human chondrocytes. Moreover, ANE treatment down‐regulated the markers of chondrocyte hypertrophy, such as collagen X, in both IL‐1β‐treated human cartilage and joint cartilage of mice with DMM surgery. Therefore, we speculate that ANE ameliorated the degeneration of articular cartilage also by inhibiting chondrocyte hypertrophy during OA development.

Although the degeneration of articular cartilage and remodelling of subchondral bone are the major characteristics of OA, other joint tissues such as the synovial membranes also actively participate in the pathogenesis of OA 47. For example, synovial membrane inflammation plays an important role in the pathophysiology of OA 48. One limitation of our study is that we did not investigate the effects of ANE on the articular synovium in OA. In addition, other signalling pathways such as JNK, and p38 MAPK signalling pathway are also involved in the pathogenesis of OA 39. More elaborate studies are also needed to explore the effects of ANE on these signalling pathways during OA progression.

In summary, for the first time, we found that ANE, a natural molecule from plants, attenuated OA progression in murine DMM model probably by suppressing IL‐1/NF‐κB pathway. Our finding raised the potential clinical application of ANE in OA therapy.

## Competing interests

The authors declare that they have no competing interests.

## Supporting information


**Figure S1** The structure of Anemonin.

**Figure S2** Screen of the optimal dose of anemonin for the treatment of DMM mice.

**Figure S3** The effects of ANE on articular cartilage in non‐DMM mice.

**Figure S4** The Primer sequences for quantitative RT‐PCR in this study.

**Figure S5** Effects of anemonin and ibuprofen on human articular cartilage.

**Figure S6** (A) Western blot analysis of MMP13, Aggrecan and phosphorylation of p65 in RCS cells treated with the combination of recombinant human IL‐1β, ANE and siRNANF‐κBp65 for 4h. β‐actin was used as a loading control. Data are expressed as the normalized fold expression relative to controls. (B‐D)The signal intensities of MMP13, Aggrecan and phosphorylation of p65 were quantified using software ImageJ (version 1.47). Data are expressed as the mean (symbols) 95% confidence intervals (error bar). *P*‐values between groups with * are less than 0.05.
