# Supplementary figures and images for: Anemonin attenuates osteoarthritis progression through inhibiting the activation of IL‐1β/NF‐κB pathway
Source: J Cell Mol Med. 2017 Jun 23;21(12):3231–43. doi: 10.1111/jcmm.13227 (PMC5706500; doi:10.1111/jcmm.13227)

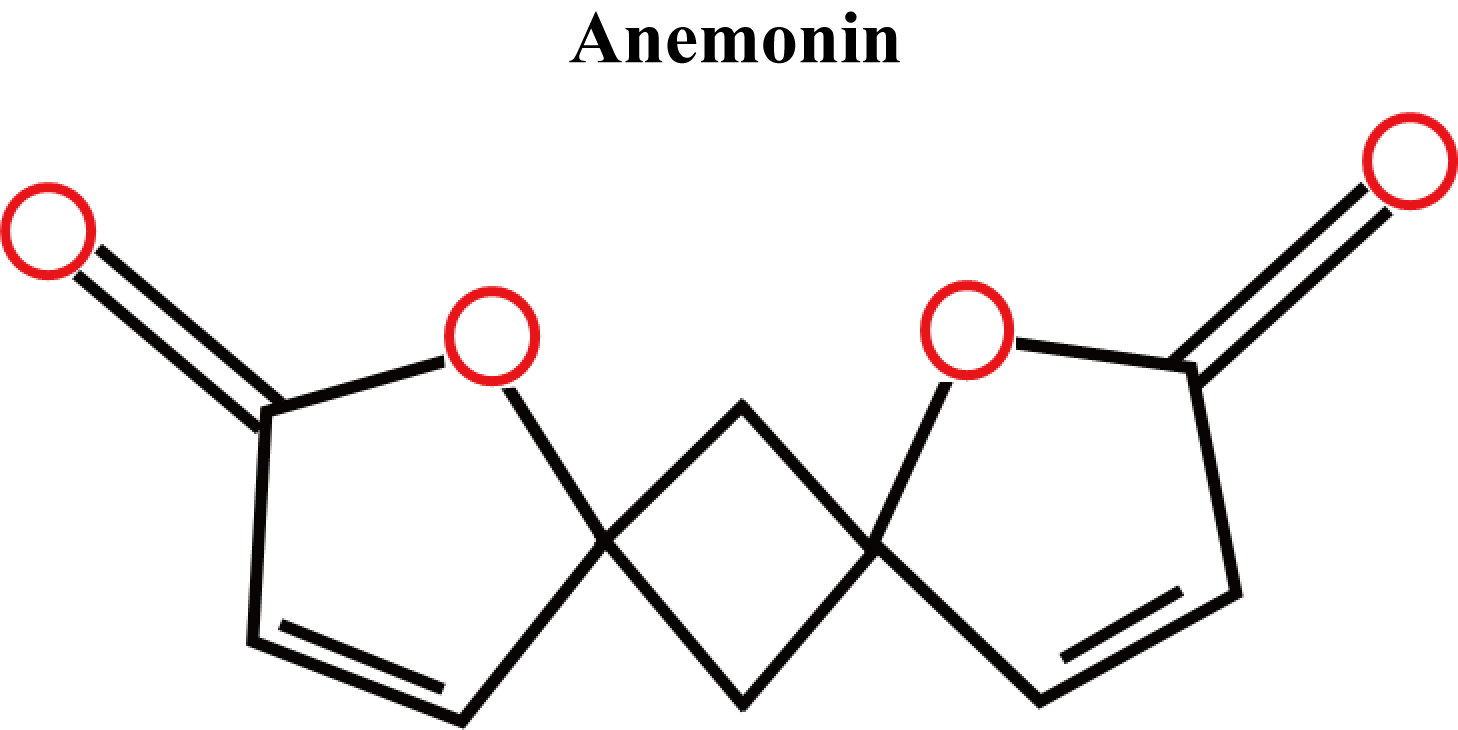

Supplement: Supplementary file 1 — Figure S1 The structure of Anemonin. [file JCMM-21-3231-s001.tif]

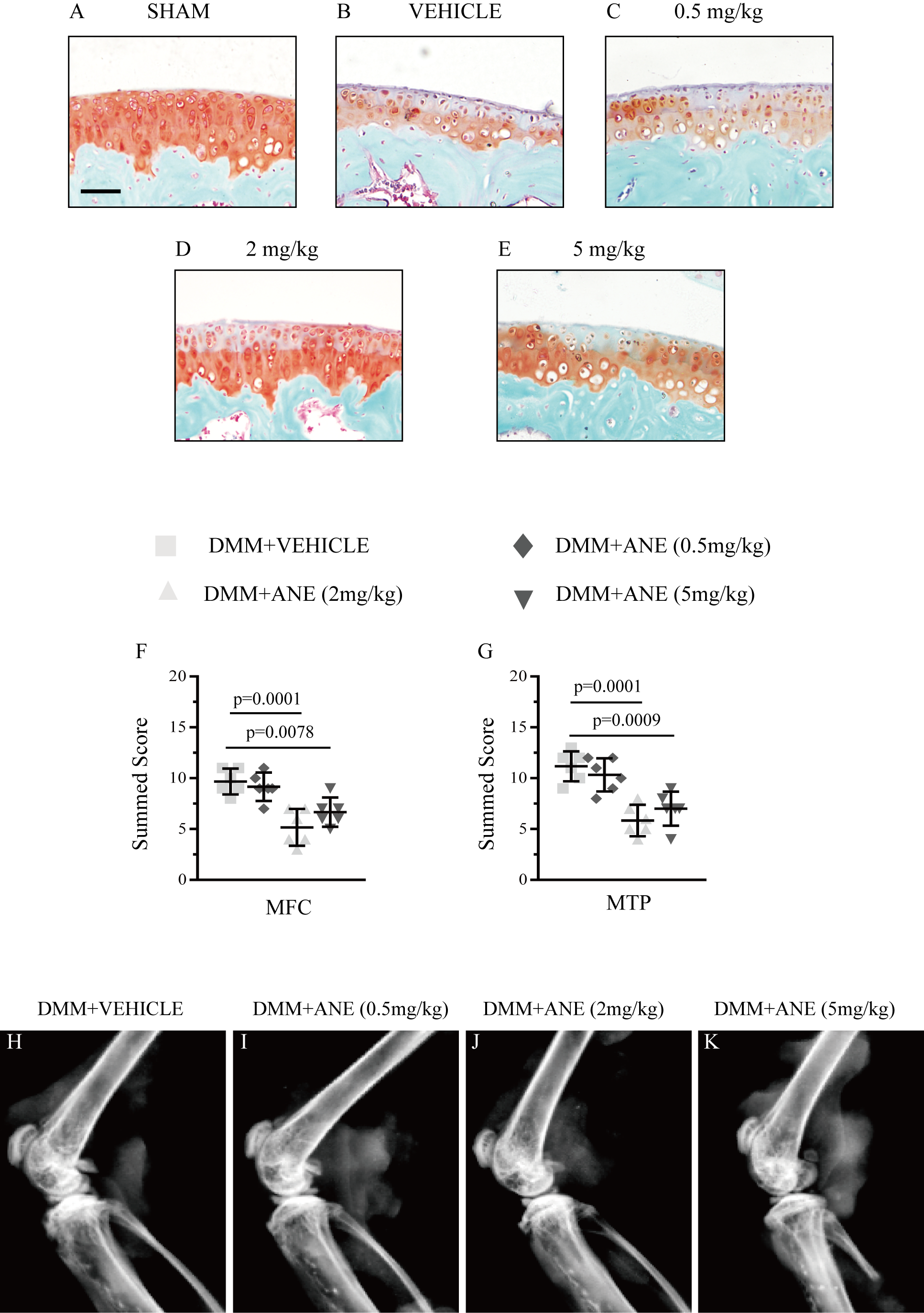

Supplement: Supplementary file 2 — Figure S2 Screen of the optimal dose of anemonin for the treatment of DMM mice. [file JCMM-21-3231-s002.tif]

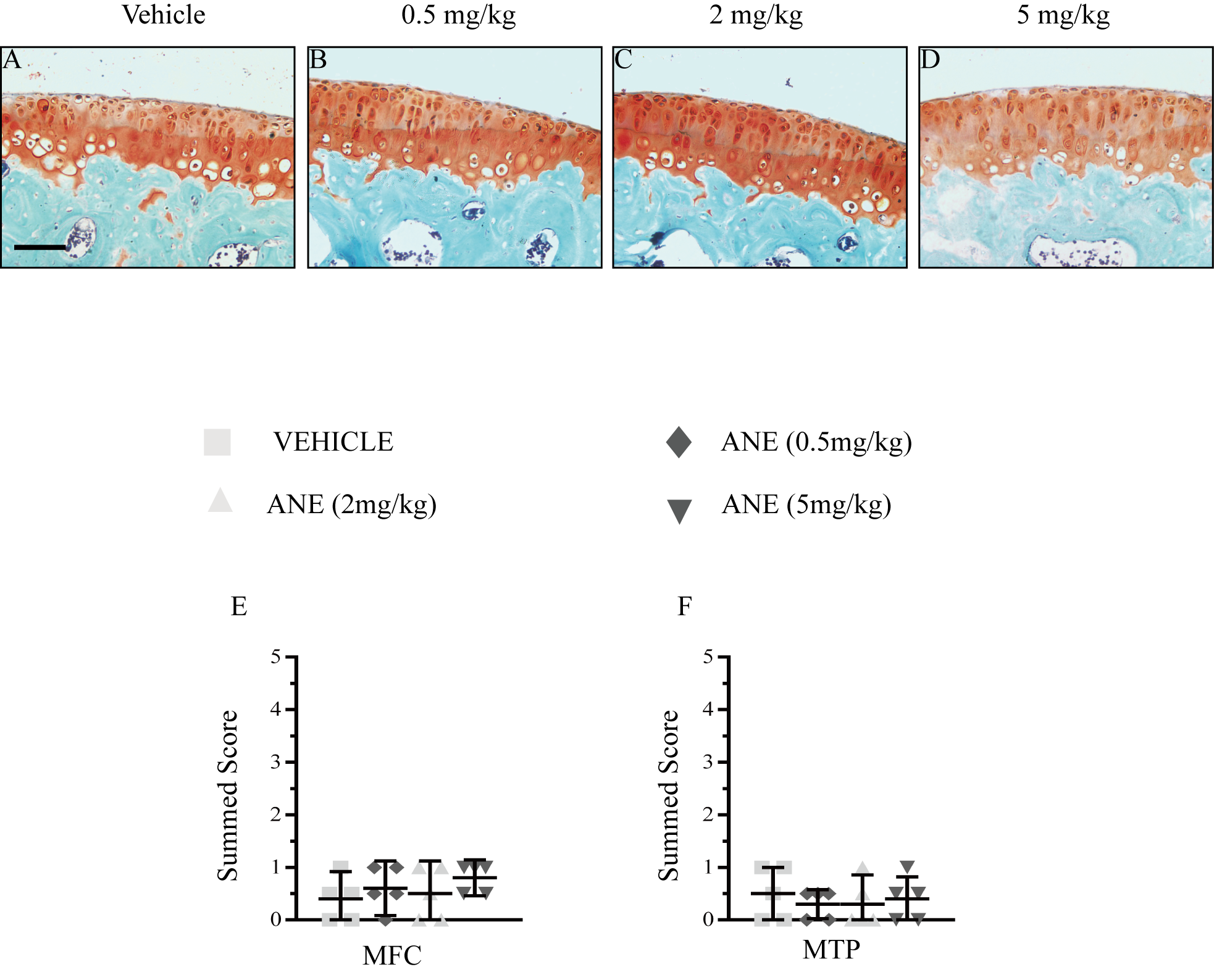

Supplement: Supplementary file 3 — Figure S3 The effects of ANE on articular cartilage in non‐DMM mice. [file JCMM-21-3231-s003.tif]

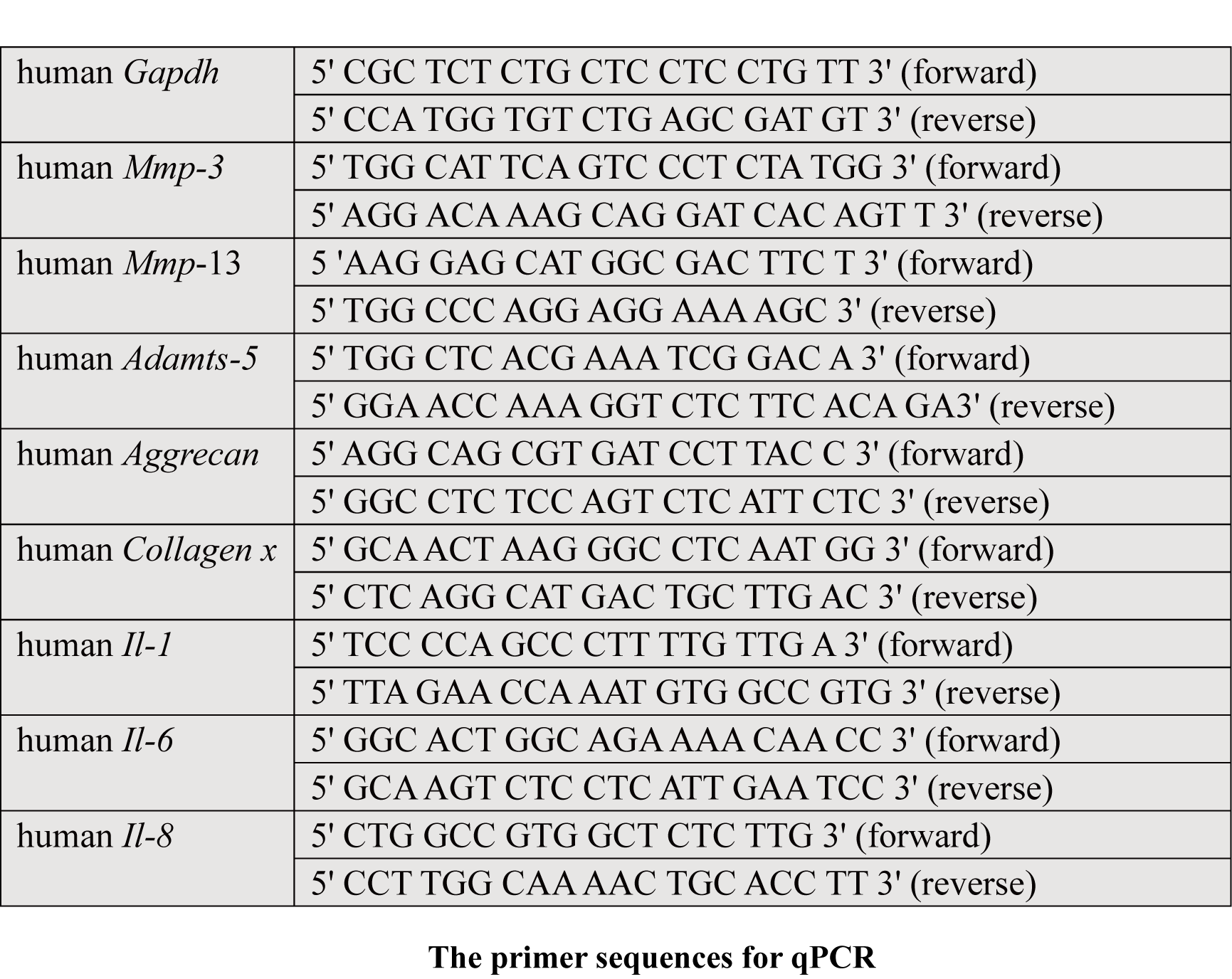

Supplement: Supplementary file 4 — Figure S4 The Primer sequences for quantitative RT‐PCR in this study. [file JCMM-21-3231-s004.tif]

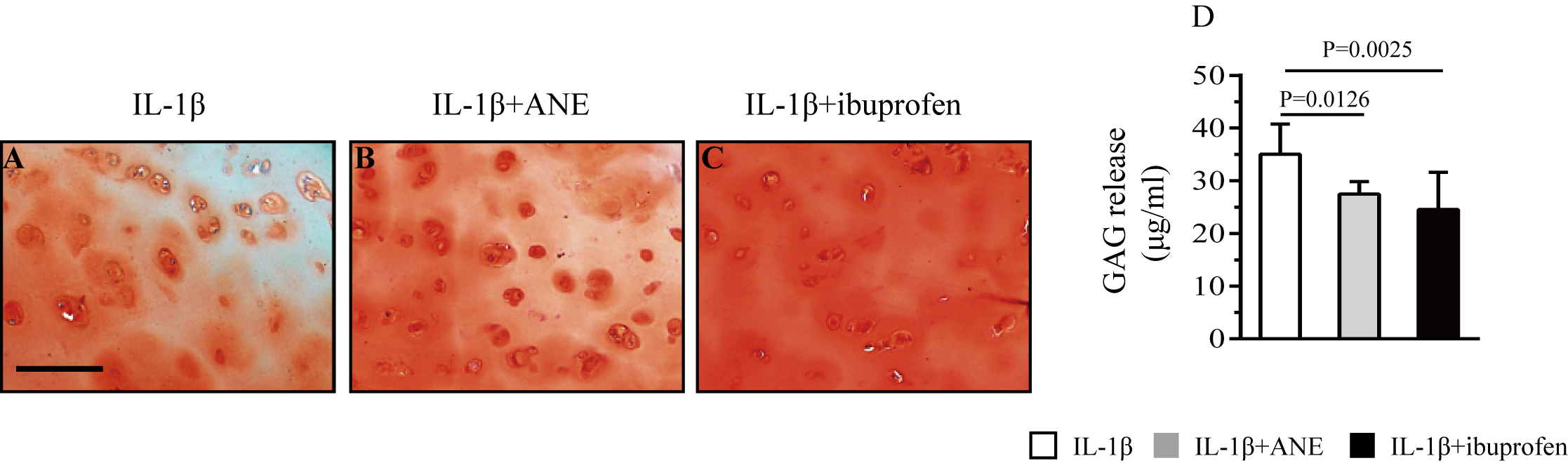

Supplement: Supplementary file 5 — Figure S5 Effects of anemonin and ibuprofen on human articular cartilage. [file JCMM-21-3231-s005.tif]

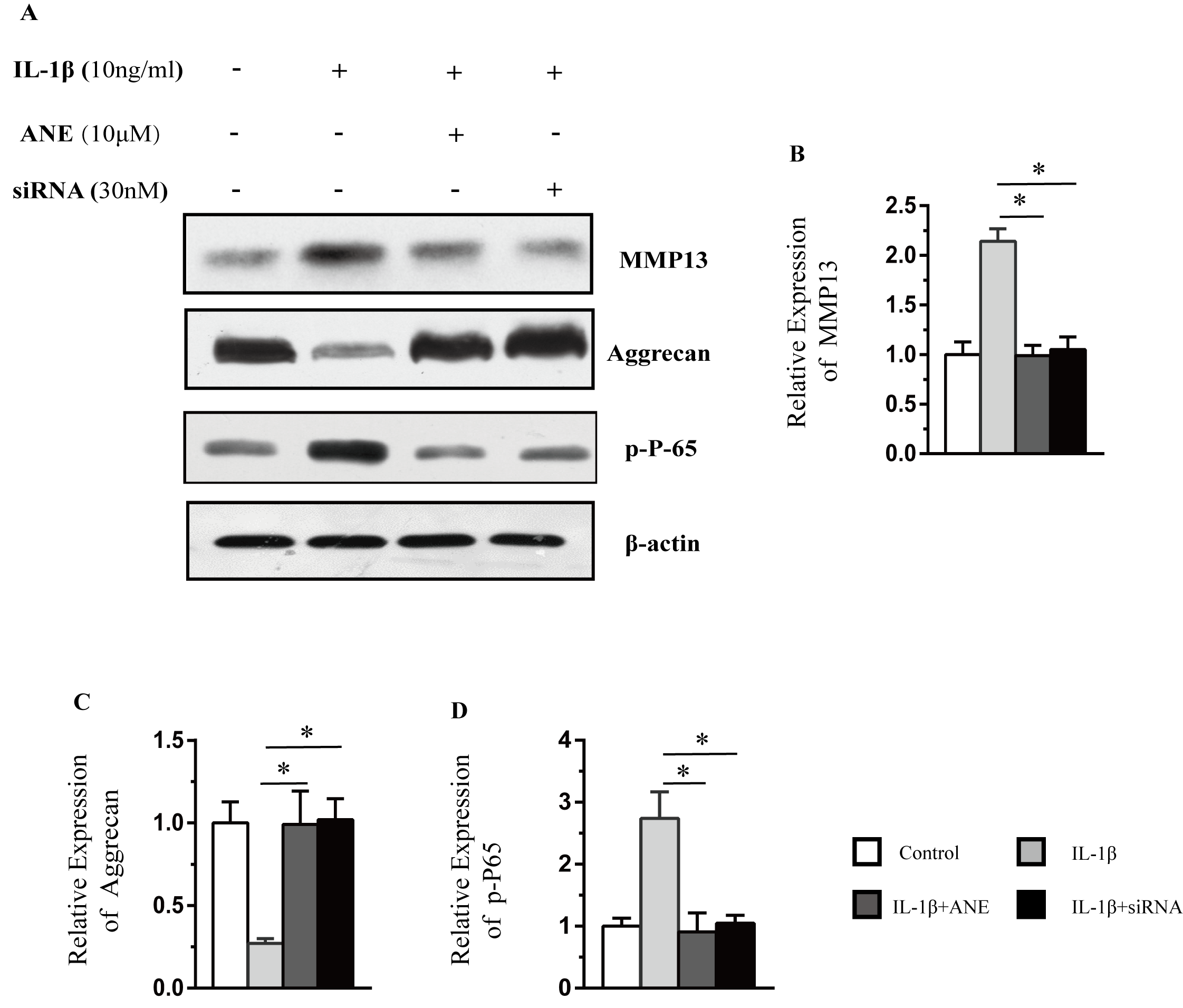

Supplement: Supplementary file 6 — Figure S6 (A) Western blot analysis of MMP13, Aggrecan and phosphorylation of p65 in RCS cells treated with the combination of recombinant human IL‐1β, ANE and siRNANF‐κBp65 for 4h. β‐actin was used as a loading control. Data are expressed as the normalized fold expression relative to controls. (B‐D)The signal intensities of MMP13, Aggrecan and phosphorylation of p65 were quantified using software ImageJ (version 1.47). Data are expressed as the mean (symbols) 95% confidence intervals (error bar). P‐values between groups with * are less than 0.05. [file JCMM-21-3231-s006.tif]
